# Insights into the Health-Promoting Properties and Nutraceutical and Food Applications of *Lonicera japonica* Thunb.

**DOI:** 10.3390/molecules31193540

**Published:** 2026-10-04

**Authors:** Shuzhen Wang, Fu Xiang, Wei Long, Lei Zhang, Wanju Zhang, Feng He, Chi-Tang Ho, Shiming Li

**Affiliations:** 1 LiShizhen Culture and Industry Research Center of Traditional Chinese Medicine, Li Shizhen College of Traditional Chinese Medicine and College of Biology and Agricultural Resources, Huanggang Normal University, Huanggang 438000, China; 2 Department of Food Science, Rutgers University, New Brunswick, NJ 08901, USA

**Keywords:** *Lonicera japonica* Thunberg, traditional Chinese medicine, phytochemistry, pharmacological activities, nutraceuticals, food application

## Abstract

*Lonicera japonica* Thunb. (*L. japonica*), an edible medicinal plant that has been widely consumed in East Asian countries, including China, Japan, and Korea, for centuries, has long been regarded as a superior herb in traditional Chinese medicine and is an economically important crop commonly used in beverages, foods, and herbal medicines. Extensive studies have demonstrated that *L. japonica* possesses a broad spectrum of health-promoting properties, including antioxidant, antibacterial, antiviral, immunomodulatory, metabolic regulatory, and anti-endotoxin activities. This review comprehensively summarizes the distribution, cultivation, quality evaluation, cultivar identification, bioactive constituents, and health-promoting effects of *L. japonica*, while also highlighting key scientific questions that warrant further investigation. Furthermore, the molecular mechanisms responsible for the major pharmacological activities of *L. japonica* flowers—including anti-inflammatory and immunomodulatory effects—are comprehensively discussed. This review provides an up-to-date scientific foundation and valuable insights to facilitate the continued development and diversified applications of *L. japonica* in the pharmaceutical, nutraceutical, and functional food industries.

## 1. Introduction

**Figure 1:**
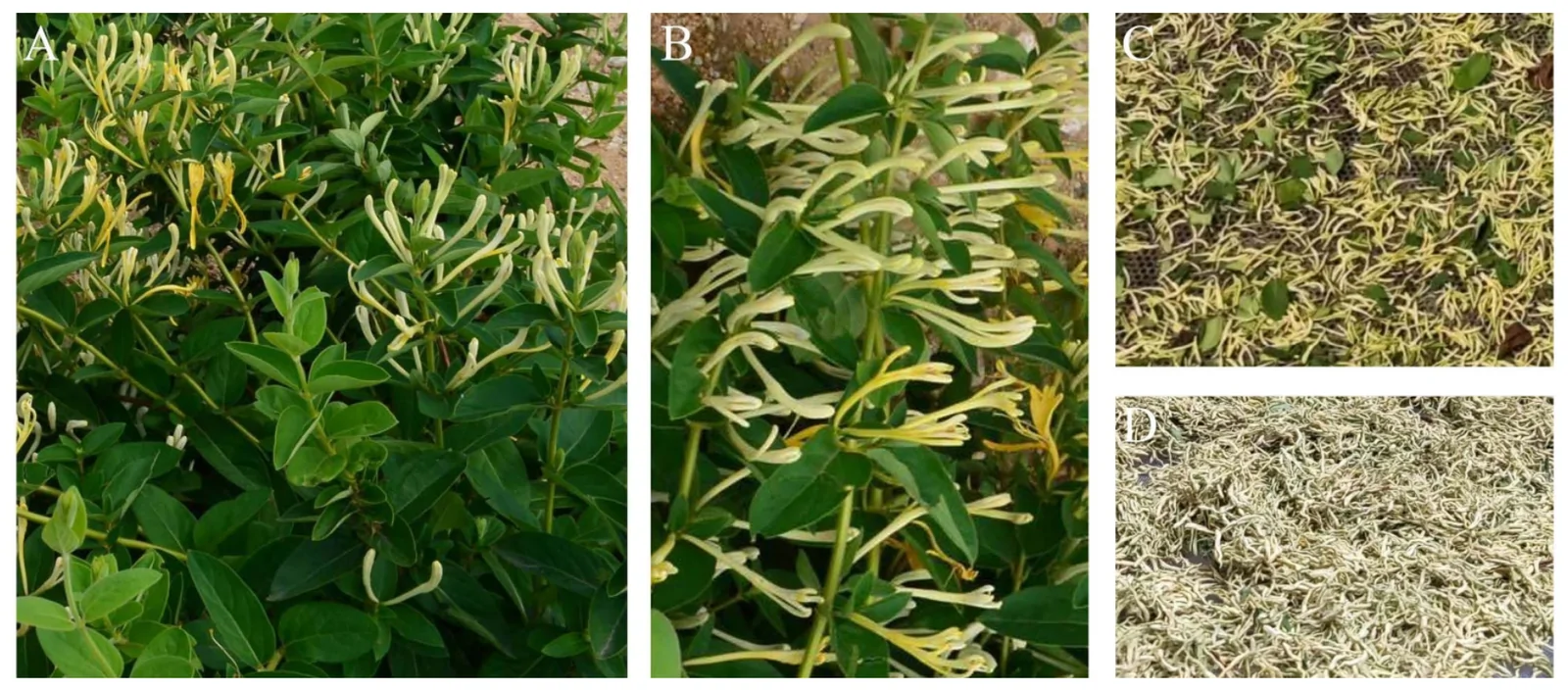
Morphological characteristics of *L. japonica* ((**A**): the whole plant; (**B**): branch with flower buds; (**C**): freshly picked flower buds; (**D**): dried flower buds).

*Lonicera japonica* Thunb. (*L. japonica*, Caprifoliaceae), commonly known as Japanese honeysuckle, Jin Yin Hua, or Ren Dong, is one of the most important medicinal plants in traditional Chinese medicine (TCM). Though native to China, Japan, Korea, and Manchuria, *L. japonica* has been introduced to many countries with a list of heterotypic and homotypic synonyms, including *Nintooa japonica* (Thunb.) Sweet, *Caprifolium japonicum* (Thunb.) Dum. Cours., *Caprifolium chinense* S. Watson ex G. Don, *L. chinensis* P. Watson, and *L. aureoreticulata* T. Moore (https://powo.science.kew.org/taxon/urn:lsid:ipni.org:names:148850-1, accessed on 20 July 2026). It has been documented in numerous classical Chinese medical texts, including *Shen Nong Ben Cao Jing* and *Ming Yi Bie Lu*, and classified as a “top-grade” herb because of its effectiveness in mitigating febrile diseases and inflammatory conditions [1,2]. In TCM, the aerial parts of *L. japonica* have traditionally been used to treat conditions associated with infection and inflammation. Among them, *L. japonicae* flos (the dried flower buds or newly opened flowers) and *L. japonicae* Caulis (the dried stems, known as *Ren Dong Teng*) are the most commonly used medicinal materials (Figure 1) [3,4]. Today, extracts of *L. japonica* are incorporated into more than 70% of Chinese patented medicines indicated for the management of inflammatory disorders and common cold-related symptoms [5]. In addition to its medicinal applications, dried *L. japonica* flowers are widely consumed in China as herbal teas and are increasingly incorporated into functional beverages and other health-promoting foods.

We searched databases including PubMed (https://pubmed.ncbi.nlm.nih.gov, accessed on 20 July 2026), Google Scholar (www. https://scholar.google.com, accessed on 20 July 2026) and SciFinder (www.cas.org, accessed on 20 July 2026), among others, for literature published between 2000 and 2026. The keyword “*Lonicera japonica*” was initially used, yielding more than 15,000 results. The search was then refined using terms related to its bioactive compounds, biological properties, and applications in foods and beverages. These terms include “phytochemicals”, “bioactives”, “biological activity”, “bioavailability”, “adverse effect”, “food application”, “beverages”, and “functiona foods”, among others. Accordingly, this review covers the major phytochemicals identified in *L. japonica*, their associated biological activities and underlying mechanisms, and the applications of *L. japonica* in foods and beverages.

### 1.1. Distribution and Cultivation of L. japonica

Geographical traceability plays a crucial role in ensuring the authenticity, quality, and consistency of medicinal plants [6]. *L. japonica*, a perennial evergreen vine characterized by its twining or sprawling habit, numerous sweetly fragrant white flowers, perennial and attractive evergreen foliage, is native to China, Japan, and Korea, and has subsequently been introduced to Argentina, Australia, Brazil, Mexico, New Zealand, and the United States [2]. In China, *L. japonica* is widely distributed throughout the Yellow River and Yangzi River basins, and extensively cultivated in several major production areas, particularly Hunan, Henan and Shandong provinces [2]. Particularly, *L. japonica* exhibits diverse patterns of organ fusion involving leaves, bracteoles, and ovaries, which is a model system for studying evolution and development of organ fusion [7]. Compared with wild populations, cultivated *L. japonica* exhibits more erect plant morphology, higher flower-to-leaf ratio, more obvious aggregation of flowers into clusters at the top of branches, increased flowering frequency, and longer floral buds [8]. In contrast, wild individuals display stronger resistance to powdery mildew than cultivated varieties [8].

Twining vine and dynamic flower coloration (transition from white to golden yellow) of *L. japonica* are also important ornamental traits. Following the first frost, *L. japonica* leaves remain photosynthetically active, accompanied by a significant increase in the accumulation of bioactive constituents [9]. Pruning branches and leaves is a common agronomic practice to promote the development of flower buds and enhance flower yield [9]. The concentrations of bioactive compounds in pruned leaves reach their highest levels in early March, indicating that this period is optimal for leaf harvest [9]. In addition, the composition of endophytic bacterial communities varies among different plant tissues but remains relatively stable within the same tissue type across different cultivation sites [10]. Environmental factors, particularly geographic origin and growing conditions, can markedly influence the accumulation of certain secondary metabolites, highlighting the importance of suitable cultivation regions for producing high-quality *L. japonica*.

### 1.2. Quality Evaluation and Identification of L. japonica

Morphological characteristics are closely associated with internal quality, as the contents of chlorogenic acid, isochlorogenic acid C, sweroside, loganin, and total phenolic acids correlate with commercial grades. Geographical origin and light conditions markedly influence flavor and the accumulation of bioactive constituents, particularly flavonoids [11]. Compared with diploid plants, tetraploid *L. japonica* exhibits higher biomass, increased phenolic acid and flavonoid contents, and greater salt tolerance [12]. In addition, endophytic microorganisms contribute to quality of *L. japonica* by enhancing antioxidant defenses and increasing tolerance to cadmium stress [13]. Macranthoidin A, macranthoidin B, dipsacoside B, and secologanic acid are the main differential components in different *L. japonica* flos samples [14,15]. In particular, sweroside, secologanoside, and secoxyloganin are markers of cultivated and wild *L. japonica* flos; lonicerin, kaempferol-3-O-rutinoside, loganin, isochlorogenic acid B, isochlorogenic acid C, secologanic acid, luteoloside, astragalin could be used to identify aerial parts of *L. japonica* [16]. Chlorogenic acid, ferulic acid, 5-*O*-caffeoylquinic acid, and caffeic acid are important components to distinguish different growth periods of *L. japonica* flos [17]. The combination of chlorogenic acid methyl ester and caffeic acid can also distinguish germplasms of *L. japonica* flos [17]. Nuclear magnetic resonance spectroscopy, infrared spectroscopy, liquid chromatography, gas chromatography, mass spectrometry, and fluorescence detection technology coupled with multicomponent quantitative analysis, chemical pattern recognition, DNA metabarcoding technology, diboronic acid-based fluorescent sensor array, and machine learning techniques, are rapid and effective tools for the successful discrimination of *L. japonica* flos and its containing formulations [15,16,17,18].

### 1.3. Important Genes in L. japonica Genome

Recent advances in multi-omics have greatly accelerated functional genomic studies of *L. japonica*. Several publicly available databases, including LjFT, LjMYB12, LoniComp, and LjaFGD, integrate genomic, transcriptomic, and functional annotation data, providing valuable resources for gene function analysis and molecular breeding [19,20]. The chromosome-level reference genome (843.2 Mb, nine pseudochromosomes), together with complete chloroplast genomes and conserved miRNAs, have established a solid foundation for species identification, evolutionary analysis, quality evaluation, and genetic improvement of *L. japonica* [21,22,23,24,25]. High-throughput transcriptomic analyses have identified numerous genes associated with secondary metabolism, stress responses, flower development, and hormone signaling [26,27,28,29]. Differentially expressed genes involved in phenylpropanoid, flavonoid, carotenoid, carbohydrate, and phytohormone metabolic pathways contribute to the biosynthesis of chlorogenic acid, luteoloside, iridoids, and flower color transition [26,27,28,29]. Polyploidization further enhances the expression of genes involved in hormone signaling, carbon metabolism, and stress resistance, contributing to improved environmental adaptability [27]. Genome-wide analyses have also identified numerous regulatory factors, including MYB, bHLH, WRKY, NAC, AP2/ERF, and bZIP transcription factor families, as well as MADS-box and DNA demethylase genes, which collectively regulate secondary metabolite biosynthesis, flower development, senescence, and stress responses [30,31,32,33,34]. In particular, LjaMYB12, LjaMYB305, 4-coumarate:CoA ligase (4CL), and chalcone synthase (CHS) have been identified as key regulators of flavonoid biosynthesis, while histone methylation, also contribute to the accumulation of chlorogenic acid, loganic acid, and luteoloside [35,36,37,38].

## 2. Bioactive Phytochemicals in *L. japonica*

*L. japonica* extracts comprise essential oils, organic acids, flavonoids, triterpenes, saponins, iridoids and inorganic elements. These compounds are biosynthesized and structurally diversified through a series of enzymatic reactions, including deglycosylation, isomerization, hydrogenation, methylation, dehydration, cyclization, glycosylation and demethylation. The phytochemical composition of *L. japonica* varies considerably among flowers (including buds), leaves and stems and is influenced by genetic background, epigenetic regulation, and developmental stage. Green flower buds are particularly enriched in caffeic acid, chlorogenic acid, sauchinone, coniferin, secoisolariciresinol diglucoside, scopolin, methyl cinnamate, and 7-hydroxycoumarin. Leaves contain higher levels of hydroxycinnamic acids and flavonoids than buds and stems. In addition, the characteristic aroma of *L. japonica* is primarily attributed to terpenoids, including monoterpenoids (e.g., geraniol and alpha-terpineol), sesquiterpenoids (e.g., farnesol, farnesal, and alpha-farnesene), triterpenoid (e.g., squalene), together with tryptophan-derivative compounds (e.g., methyl anthranilate), as well as fatty acid derivatives [39,40,41,42,43]. In relation to biological activities, these compounds exerted synergistic effects.

### 2.1. Organic Acids

**Figure 2:**
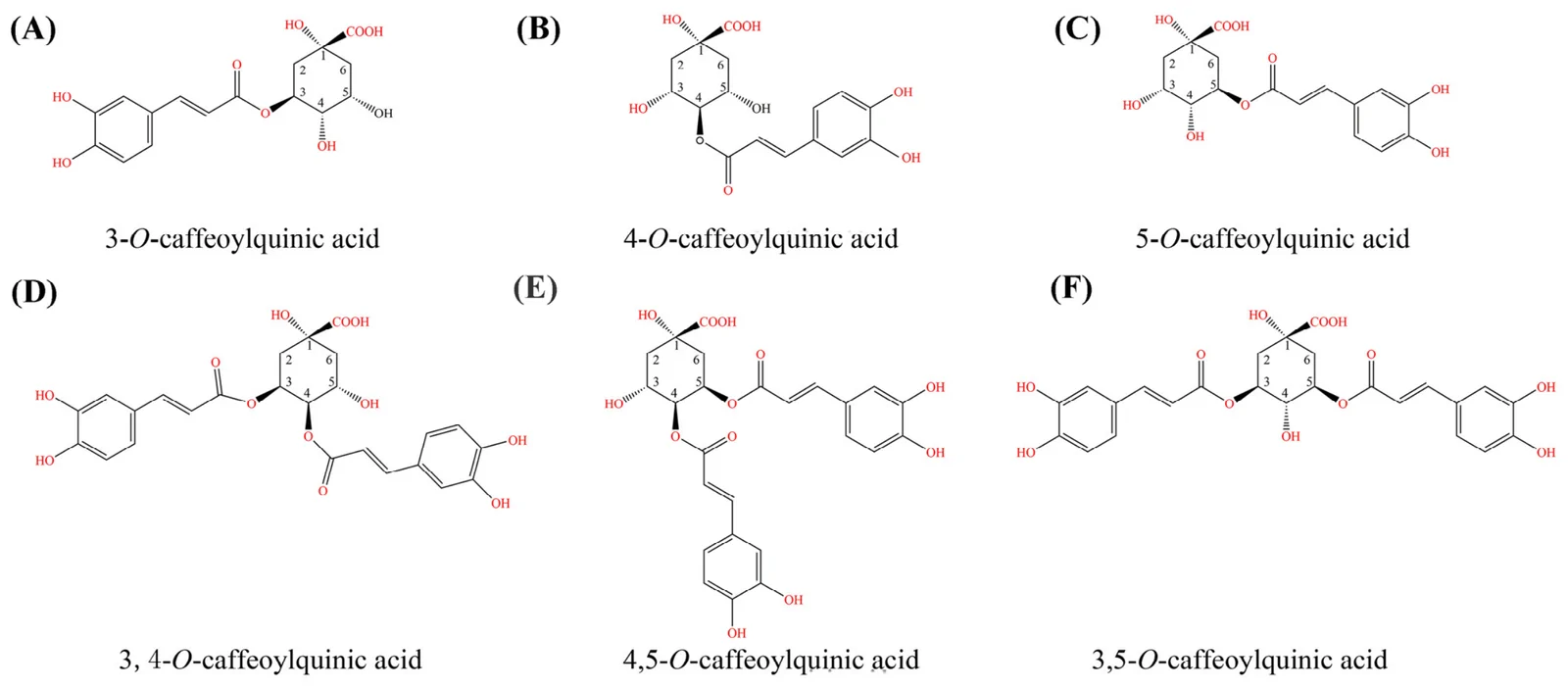
Chemical structures of main chlorogenic acids in *L. japonica* flos.

**Figure 3:**
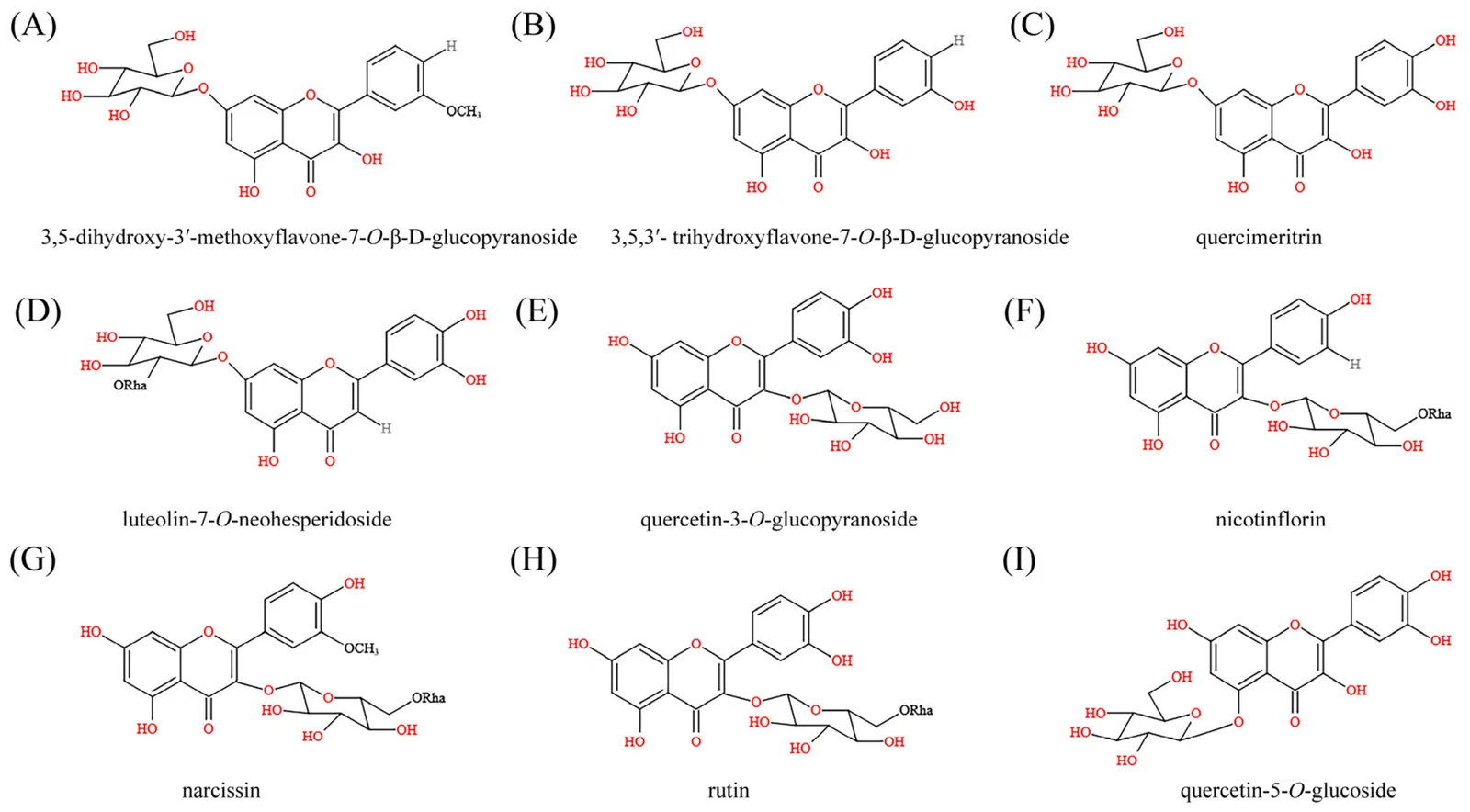
Flavonoid glycosides isolated from *L. japonica* flower buds.

Organic acids are the predominant bioactive constituents of *L. japonica* leaves and flowers, with chlorogenic acid, isochlorogenic acid, protocatechuic acid, hydroxycinnamic acids (e.g., *p*-hydroxycinnamic acid), caffeic acid, hexadecanoic acid, myristic acid, and tetraacetyl-phthalein being the main organic acids (Figure 2). A ternary deep eutectic system composed of choline dihydrogen citrate, lactic acid, and urea demonstrated superior extraction efficiency for phenolic acids from *L. japonica* [44]. During the processing of *L. japonica* flos, the contents of chlorogenic acid and dicaffeoylquinic acid isomers (3,4-. 3,5-, and 4,5-dicaffeoylquinic acid) change significantly [45]. In the Chinese Pharmacopeia, chlorogenic acid is designated as an official quality indicator for *L. japonica* quality. Furthermore, the contents of chlorogenic acid and luteolin (a flavone shown in Figure 3) gradually decrease from the green bud stage to the white flower stage [34]. Chlorogenic acid biosynthesis in *L. japonica* is regulated by several key enzymes, including shikimate kinase 1, cinnamate 4-hydroxylase, and hydroxycinnamoylcoenzyme A shikimate/quinate hydroxycinnamoyltransferase were involved in the synthesis pathway of chlorogenic acid, all of which are up-regulated in flowers [30]. Salinity stress also promotes chlorogenic acid accumulation, with concentrations in leaves being increased by 145.74% and 50.34% after 30 days treatment of 150 and 300 mM NaCl, respectively [46]. Similarly, soil salinity could elevate chlorogenic acid concentrations in honeysuckle flower buds by 22.42–25.14% [46]. Furthermore, phenolic acid glycosides are particularly abundant in flower buds, whereas caffeic acid is present at relatively low levels in flower bud but accumulates predominantly in stems and leaves of *L. japonica* [39,47].

### 2.2. Essential Oils

Essential oils are primarily distributed in the aerial parts of *L. japonica*, including fresh and dried flowers, leaves, and stems (vines). Their main constituents include linalool, hexadecanoic acid, octadecadienoic acid, methyl linolenate, methylpalmita, propylbenzene, ethyl benzene, benzoic aldehyde, translinalool, isophytol, cyclohexano, cyclohexylisooxalic ester, methylcyclohexane, n-hexadecanoic acid, ethyl palmitate, (*Z*)-jasmone, (*Z*)-jasmin lactone, methyl jasmonate, methyl epi-jasmonate, and dihydrocarveol [2]. The composition of essential oils varies markedly among different developmental stages and plant tissues. In the flowers of *L. japonica*, the predominant constituents are aromadendrene, linalool, geraniol, hexadecanoic acid, together with various aldehydes, ketones, acids, and esters. For example, the content of alkanes was highest in buds and lowest in leaves; whereas levels of aldehydes was highest in leaves but lowest in buds [2]. During flower development from bud stage to fully opened golden flowers, the level of alkanes, alcohols and ketones gradually increase, while those of organic acids and esters progressively decline [2]. Particularly, the chemical composition and yield of essential oils are influenced by multiple factors, including the medicinal plant part used, harvest stage, extraction method, and post-harvest processing conditions.

### 2.3. Flavonoids

The distribution of flavonoids in *L. japonica* is tissue-specific. Flavones are predominantly found in the leaves (0.65–4.31%), flavonols mainly accumulate in flower buds, whereas acylated flavonol glycosides are primarily present in flowers [2,39]. Structures of some flavonoids from *L. japonica* are illustrated in Figure 3. For instance, several flavonoid glycosides, including luteoloside and luteolin-7-*O*-[α-*L*-arabinopyranosyl-(1→6)]-β-*D*-glucopyranoside, have been predominantly identified in *L. japonica* leaves [48]. In addition, different solvent fractions yielded distinct flavonoid profiles. Luteolin, quercetin, and kaempferol were isolated from ethyl acetate (EtOAc) fraction; quercetin-3-*O*-β-*D*-glucoside, hyperoside, luteolin-7-*O*-α-*D*-glucoside, and luteolin-7-*O*-α-*D*-galactoside were obtained from the n-butanol extracts; whereas corymbosin and 5-hydroxy-3′,4′,7-trimethoxylflavone were isolated from chloroform extracts [49]. Biosynthesis and accumulation of *L. japonica* flavonoids are closely associated with developmental stage, flower color, and enzymatic regulation. Flavonoid glycosides such as luteolin 7-glucoside were positively correlated with astringency, suggesting that flavonoid glycosylation may contribute to the sensory characteristics of *L. japonica* [50]. Flower color variation is also closely linked to flavonoid metabolism: the purple-flowered cultivar of *L. japonica* accumulated more than 10-fold higher levels of anthocyanins than the green-flowered cultivar, while the content of cynaroside was significantly more abundant in red- than in green-flowered honeysuckle [51,52]. During flower development, simple phenylpropanoids and flavonoids accumulated preferentially at early stages, whereas their downstream derivatives increased at late stages [53]. At molecular level, glycosyltransferases may participate in the downstream modification and diversification of flavonoids [54]. Moreover, exogenous chlorogenic acid treatment consistently enhanced flavonoid accumulation in *L. japonica* leaves, suggesting metabolic crosstalk between phenolic acid and flavonoid pathways [55]. Enzymatic studies further demonstrated that recombinant cytochrome P450 proteins, in the presence of NADPH, directly converted eriodictyol, naringenin, and liquiritigenin to corresponding flavones, highlighting the role of P450-mediated hydroxylation in flavonoid diversification [56].

### 2.4. Terpenoids

**Figure 4:**
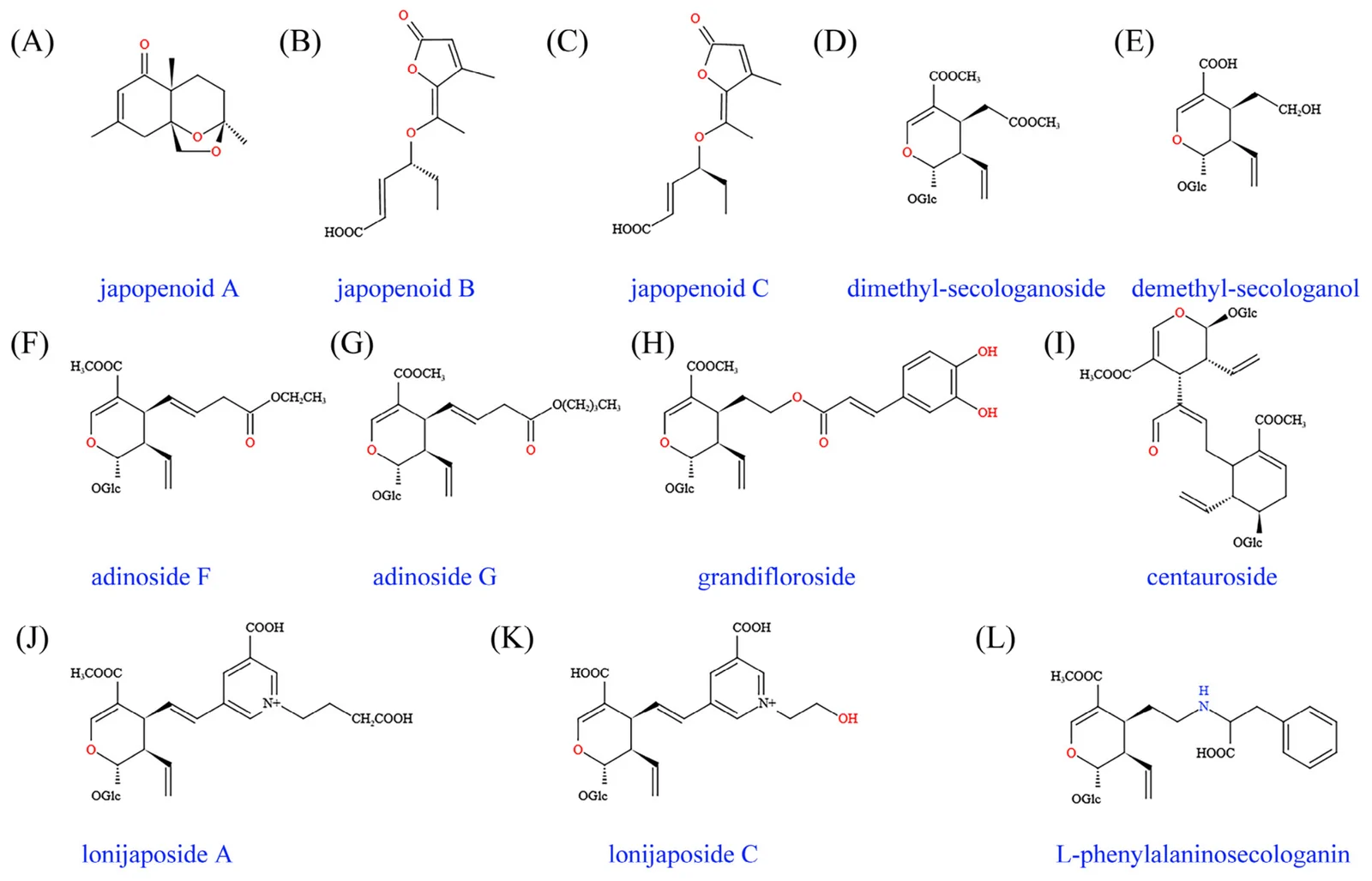
Chemical structures of monoterpenoids identified in *L. japonica* flower buds.

Iridoids and their glycosides exhibit diverse biological activities, including antitumor, antivirus, anti-inflammatory, antioxidant activity, hypoglycemic, and hepatoprotective effects. They also contribute to the characteristic flavor of *L. japonica* and serve as important quality markers [50]. A wide range of iridoid glycosides, including sweroside, 7-*O*-ethyl sweroside, 7-epi vogeloside, secoxyloganin, secoxyloganin 7-butyl ester, 7-dimethyl-secologanoside, centauroside, secologanic acid, secologanin, secologanin dimethyl acetal, kingiside, vogeloside, epi-vogeloside, dehydrornorronisi, ketologanin, 7α-morroniside, 7β-morroniside, secologanoside, and 12 pyridinium inner-salt alkaloids lonijaposides (A-L), have been isolated from the flower buds of *L. japonica* [57,58]. Chemical structures of monoterpenoids identified in *L. japonica* flower buds were as Figure 4. In addition, japonicaside C, a rare heterodimeric terpenoid derivative biogenetically originated from a guaiane-type sesquiterpenoid and a secoiridoid glucoside, was isolated from the flower buds of *L. japonica* [59]. Particularly, the secoiridoid glycosides loniceracetalide A and loniceracetalide B only existed in dried flower buds [60]. Iridoid glycosides, including secologanolic acid, (*Z*)-aldsecologanin, (*E*)-aldosecologanin, L-phenylalaninosecologanin, 7-*O*-(4-β-D-glucopyranosyloxy-3-methoxy-benzoyl) loganin, and 6′-*O*-(7α-hydroxyswerosyloxy) loganin were isolated from the stems and leaves of *L. japonica* [61]. Secologanoside, sweroside, and secoxyloganin were isolated from the EtOAc fraction of *L. japonica* extracts [49]. Terpenoid biosynthesis was promoted by transketolase-contained regulatory circuit in late stages of *L. japonica* flower development [62]. Yellow flower petals contained much higher levels of carotenoids than green bud petals and white flower petals, with α-carotene, zeaxanthin, violaxanthin and γ-carotene being the major carotenoid compounds [28].

### 2.5. Saccharides

*L. japonica* polysaccharides (LJP) demonstrated anti-inflammatory, antioxidant, immunomodulatory, anti-Alzheimer’s, anti-diabetic, and anti-cancer effects (Table 1) [63,64,65,66,67,68,69,70,71,72,73,74,75,76]. A homogeneous polysaccharide fraction, LJ-02-1, with a molecular weight of 54 kDa was isolated and purified from *L. japonica* flowers using DEAE-cellulose and Sephacryl S-200HR column chromatography and exhibited antipancreatic cancer activity [77]. The LJ-02-1 consisted primarily of rhamnose, galacturonic acid, galactose, and arabinose in a molar ratio of 10.77:7.88:15.45:65.89 [77]. Structural analysis indicated that LJ-02-1 has a rhamnogalacturonan backbone, partially substituted at C-4 of rhamnose, with branches containing terminal and 1,4,6-linked β-d-Galp, and terminal and 1,5-linked α-l-Araf [77]. In addition, 1-deoxy-D-xylulose-5-phosphate accumulated in early stages and was upregulated in late stages [53].

### 2.6. Proteins

Proteins involved in oxidative pentose phosphate pathway, signaling, hormone metabolism, and transport were highly enriched through a gel-free/label-free proteomic technique coupled with combinatorial peptide ligand libraries and polyethylene glycol fractionation [78]. The abundance of hydroxymethylbutenyl 4-diphosphate synthase was particularly high in immature flower buds of *L. japonica* [78]. Following UV exposure, proteins associated with glycolysis, TCA cycle/organic acid transformation, carbohydrate metabolism, oxidative pentose phosphate pathway, stress response, secondary metabolism, hormone metabolism, and mitochondrial electron transport increased during flower opening [79].

### 2.7. Other Elements

Trace elements, including Fe, Mn, Cu, Ca, Zn, Mo, Ba, Ni, Cr, Pb, V, Co, Li, Ti, Sr, Mg, As, Se, Mo, Cd, and Hg, have been identified in *L. japonica* [80]. In particular, total arsenite concentrations ranged from 275 to 635 μg/kg determined by inductively coupled plasma mass spectrometry, with arsenic species occurring in the following order: arsenite > dimethylarsinic acid > arsenobetaine > monomethylarsonic acid [81]. Moreover, 5′-*O*-methyladenosine, 2′-*O*-methyladenosine, and adenosine have been identified in the aqueous extract of *L. japonica* flower buds [32,80]. The major volatile organic compounds in *L. japonica* flower include leaf alcohol, (*E*)-2-hexen-1-ol dimer, 2-octyn-1-ol, and (*E*)-3-hexen-1-ol [82]. Phenylpropanoids accumulated during the early stages of flower development, likely associated with enhanced glycolysis; whereas terpenoids biosynthesis was promoted by a transketolase-associated regulatory circuit during late stages [53]. Saponins in *L. japonica* are predominantly of the oleanane and hederagenin types, with monodesmosides exhibiting stronger hemolytic activity than bisdesmosides [2].

## 3. Health-Promoting Activities

*L. japonica* has been listed in Pharmacopeia of the People’s Republic of China since 1995. More than 500 prescriptions containing *L. japonica* components have been documented (http://www.zysj.com.cn, accessed on 20 July 2026) and are used for the management of severe respiratory syndromes, H1N1 influenza, hand-foot-and-mouth disease, fever, headache, depression, and epidemic diseases owing to its antibacterial, antiviral, anti-endotoxin, anti-inflammatory, hypolipidemic, hepatoprotective, cytoprotective, antioxidant, anti-infectious, and antipyretic effects [39,83,84,85]. For example, the combination of *L. japonica* flos and *Forsythiae fructus* exhibits typical antibacterial, antiviral, antitumor, anti-inflammatory, and antioxidant activities [86]. Among its constituents, chlorogenic acid has outstanding pharmacological activities.

### 3.1. Regulation of Microbial Growth

#### 3.1.1. Antiviral Activity

*L. japonica*, particularly its flower buds, has been extensively used in traditional medicine for the prevention and treatment of viral infections in humans and animals. Reported antiviral targets include H1N1 (Swine) influenza A virus, respiratory syncytial virus, parainfluenza virus, A/Beijing/32/92 (H3N2), oseltamivir-resistant influenza strains, coxsackievirus B3 and B5, severe acute respiratory syndrome coronavirus-2 (SARS-CoV-2), adeno-associated virus 7, cytomegalovirus, human adenovirus III, hepatitis B virus (HBV) as well as *Mycoplasma gallisepticum* (Table 2) [87,88,89,90,91,92,93,94,95,96,97,98,99]. Several herbal combinations containing *L. japonica* have also demonstrated antiviral or anti-infective activities, particularly against respiratory infections. These include *L. japonica* flos-*Forsythiae Fructus*, *Ostericum koreanum*-*L. japonica*-*Angelica gigas* Nakai-*Clematis manshurica-*Phellodendron amurense (6:4:4:4:3), *L. japonica* flos-*Forsythiae fructus*-chito-oligosaccharid, *L. japonica* dried flower-*Anemarrhena asphodeloides* roots, *Gardenia Jasminoides* fruits-*L. japonica* flower buds-*Artemisia annua* aerial part [86,100,101,102].

Extracts of *L. japonica* flower and Huangqi exerted antiviral properties through multiple mechanisms, including upregulation of microRNAs (*let-7a*, *miR-148b*, and *miR-146a*) and interferon (IFN)-γ, suppression of pro-inflammatory cytokines (IL-6, IL-17, TNF-α, IL-1ß), prevention of viral attachment and internalization, reduction in SARS-CoV-2 Mpro activity, inhibition of SARS-CoV-2 spike protein binding to angiotensin-converting enzyme 2 (ACE2), suppression of ACE2 and epidermal growth factor receptor (EGFR) tyrosine kinase, inhibition of antigen secretion and DNA replication, modulation of HBx protein expression for viral replication, upregulation of the nuclear transcriptional factors PGC1α and PPARα, downregulation of nucleoprotein expression, and inhibition of neuraminidase activity to block release of newly formed virus particles from infected cells [103,104,105]. *L. japonica* flos also alleviated respiratory syncytial virus (RSV) infection by improving the lung index and modulating IFN-γ and IL-6 levels [106].

Main active components in *L. japonica* including hlorogenic acid, beta-sitosterol, caffeoylquinic acids, caffeic acid derivatives, kaempferol, luteolin, methyl caffeate, 2′-*O*-methyladenosine, isochlorogenic acid B, isochlorogenic acid C, 3,4,5-tri-*O*-caffeoylquinic acid methyl ester, neochlorogenic acid, secoxyloganin, baicalin, forsythia glycosides A, quercetin, isoquercitrin, astragaloside IV, rutin, 4,5-di-*O*-caffeoylquinic acid, sweroside, cryptochlorogenic acid, and loganic acid were involved in the pathways of purine metabolism, linoleic acid metabolism, and alpha-linolenic acid metabolism via regulating NF-κB, PI3K/AKT/mTOR, MAPK, TNF, FoxO, AGE-RAGE, and Toll-like receptor signaling pathway [106,107,108,109]. In particular, Phase II reactions like sulfation, glucuronidation, methylation, along with Phase I reactions such as dehydroxylation, hydrogenation, hydrolysis, N-heterocyclization, were the main metabolic processes affected by the active substances of *L. japonica* in vivo [110].

#### 3.1.2. Regulation of Bacterial Growth

The growth of *Salmonella* spp., *Shigella* spp., *Escherichia coli*, Sarcina luteus, *Bacillus cereus* ATCC14579, *Bacillus subtillis*, *Aeromonas hydrophila*, *Streptococcus mutans*, *Citrobacter rodentium*, *Canidia albicans*, *Micrococcus luteus*, *Enterococcus*, *Pseudomonas aeruginosa*, *Staphylococcus aureus*, and *Cronobacter sakazakii* can be inhibited or otherwise affected by *L. japonica* extracts (Table 2) [52,111,112]. LJP increased the abundance of intestinal probiotics (*Bifidobacterium* and *Lactobacilli*) in a colitis ulcerative mouse models and partially restored gut microbiota composition in aged mice by adjusting Firmicutes/Bacteroidetes ratio at the phylum level and increasing the relative abundances of Lactobacillaceae and Bifidobacteriacesa [113,114]. Similarly, *L. japonica*-*Radix Pueraria* treatment increased the relative abundance of genus *Lactobacillus* within the phylum Firmicutes [115].

Several *L. japonica*-based nanomaterials (silver and copper) and herbal combinations (flavone hyperoside with β-lactams/quinolones) have also demonstrated antibacterial activity [97,116]. Water and ethanolic extracts of *L. japonica*, containing chlorogenic acid, caffeic acid, lonicerin, luteolin, quercetin, kaempferol, 3,4-dicaffeoylquinic acid, iridoid glucosides (7-*O*-ethyl sweroside, secologanin dimethylacetal, adinoside F, (*7R*)-secologain *n*-butyl methyl acetyl and adinoside G), 7-acetyl-8,9-dihydroxy thymol, and 7,8-dihydroxy-9-buyryl thymol, could induce bacterial death through multiple mechanisms, including binding to the outer membrane with negative surface charge; interacting with active site of alginate secretion protein to reduce alginate secretion and biofilm formation, regulating the expression of target genes; disrupting GbpC protein function; impeding adhesin P1 protein; inhibiting ribosome assembly and peptidoglycan biosynthesis; blocking autoinducer II sensing; downregulating the expression of quorum sensing and hemolysin-related genes; inhibiting response regulator transcriptional factors; inducing cell wall lysis; compromising cell membrane integrity; down-regulating glycosyltransferase activity; decreasing the production of water-insoluble glucans; and interfering with phospholipid biosynthesis [96,97,117,118,119,120]. Particularly, chlorogenic acid exhibited stronger bacteriostatic activity against Gram-negative bacteria than against Gram-positive bacteria.

#### 3.1.3. Acanthamoebicidal and Antifungal Effects

Ethyl acetate, aqueous, and butanol fractions of dried flower buds of *L. japonica* exhibited acanthamoebicidal effects and antifungal activities against *Aspergillus niger* and *Candida albicans* (Table 2) [2,87,88,89,90,91,92,93,94,95,96,97,98,99]. In particular, the ethyl acetate fraction of dried *L. japonica* flower buds (1.5 mg/mL) reduced trophozoite viability by 48.9%, 49.2%, and 33.7% after 24, 48, and 72 h of treatment, respectively [121]. Notably, chlorogenic acid (1 mg/mL) reduced the cysts/trophozoite ratio by 100%, 84.0%, and 72.3% after 24 h, 48 h, and 72 h, respectively [121,122].

### 3.2. Antioxidant and Anti-Aging Activities

The active ingredients of *L. japonica* can dose-dependently improve the activity of superoxide dismutase (SOD), catalase, and glutathione peroxidase; decrease malondialdehyde (MDA) levels in the serum, liver and brain; scavenge hydroxyl, DPPH, and ABTS radicals; restore acetylcholinesterase levels in the brain; alleviate liver tissue damage; and enhance the degradation of hazardous substances and the biosynthesis of flavones and flavonols (Table 3 and Table 4) [123,124,125,126,127,128,129,130,131,132,133,134,135,136,137,138,139,140,141,142,143]. Water extracts of *L. japonica* flower buds exhibited stronger antioxidant activities than ethanol or supercritical fluid (CO_2_) extracts, which were attributed to their higher contents of polyphenols, flavonoids, chlorogenic acid, and luteolin-7-*O*-glucoside [84]. In addition, silver nanoparticles, synthesized using *L. japonica* leaves, as well as luteolin-Cu^2+^ and luteolin-Fe^3+^ complexes, exhibited excellent antioxidant properties [144,145]. Cookies supplemented with a 4% level of *L. japonica* extracts containing 202.65 μg/g chlorogenic acid showed strong antioxidant activity, with DPPH free radical scavenging activity (63.71 μg Vc/g), ABTS free radical scavenging activity (415.10 μg Vc/g), and ferric-reducing power equivalent to 169.58 μg Vc/g [146]. 

Crude polysaccharides from *L. japonica* (CPLJ) showed anti-aging activity in *Caenorhabditis elegans*, extending the mean lifespan by 13.97% and improving physical fitness, as evidenced by increases in motility and pharyngeal pumping rates of 40.92% and 25.72%, respectively [63]. CPLJ also activated stress-responsive genes and the antioxidant defense system, while reducing lipofuscin accumulation by 38.9%, without adversely affecting body length and fecundity [63]. Furthermore, CPLJ regulated the expression of *daf-16*, thereby modulating the expression of downstream genes, including *sod-3*, *gst-4* and *hsp-16.2* [63]. Ethanol extract of *L. japonica* (500 μg/mL) treatment increased the mean lifespan of *C. elegans* by more than 21.87% and significantly improved age-associated physiological functions [99]. These effects were associated with reduced ROS levels and fat accumulation via upregulating stress-inducible genes (*hsp-16.2*, *sod-3* and *mtl-1*), regulating expression of the D1086.10 protein, delaying amyloid β (Aβ)-protein-induced paralysis, increasing mRNA expression of the autophagy gene *bec-1*, and decreasing the expression of the autophagy substrate p62 protein by more than 40.0% [99].

Particularly, chlorogenic acid, alkaloid, secoiridoid glycoside, flavone hyperoside, de-esterified oligogalacturonides, caffeoylquinic acids, pectin fraction, rutin, isoquercetin, luteolin, eugenol, neochlorogenic acid, 3,4-*O*-dicaffeoyl quinic acid, biflavonoid japoflavone D, 3,5-*O*-dicaffeoyl quinic acid, 4,5-*O*-dicaffeoyl quinic acid, isochlorogenic acid A, isochlorogenic acid B, isochlorogenic acid C, cynaroside, 4,5-di-*O*-caffeoylquinic acid methyl ester, isoquercitrin, and luteoloside have been identified as the main active constituents corresponding to antioxidant activity and anti-aging activities. These compounds may alleviate cellular oxidative injury by reducing Bax/Bcl-2 and Bak levels; suppressing MAPKs phosphorylation; promoting AKT phosphorylation; enhancing the expression of heme oxygenase-1 (HO-1) and NAD(P)H quinone dehydrogenase; and increasing cell survival via regulating Keap1/Nrf2 signaling pathways [147,148,149,150,151,152,153,154,155,156,157].

### 3.3. Immunomodulatory Activity

Inflammatory diseases are often associated with excessive inflammatory responses resulting from the accumulation of pro-inflammatory mediators and cytokines produced by activated macrophages. Water, ethanol, ethyl acetate, and n-butanol extracts of *L. japonica* (caulis and flower) have been traditionally used to alleviate various inflammatory conditions, including allergic rhinitis, ear and toe edema, skin hyperplasia, atopic dermatitis, arthritis, auricle swelling, gastric inflammation, rheumatoid arthritis, fever, inflammatory bowel disease, ankle joint swelling, gouty arthritis, urinary bladder contraction, lung inflammation, respiratory failure, kidney inflammation, bone erosion, and osteophyte formation in joints [158,159,160,161]. The efficacy of *L. japonica* alone in restoring immune homeostasis is unsatisfactory. In contrast, herbal formulations and derived products, such as *L. japonica-Rosae Multiflorae Fructus*, *L. japonica*-*Citri Reticulatae Pericarpium* Polyphenolic extract, *L. japonica-Forsythiae Fructus*, and 1.0–10.0 nm carbon dots derived from *L. japonica* flower buds, exhibited enhanced immunomodulatory and anti-inflammatory activities (Table 4). These effects included increasing food intake and body weight; enhancing overall immune function; alleviating atrophy of lymphoid organs (thymus and spleen); promoting proliferation of macrophage and T lymphocytes (CD4+ and CD8+); inhibiting abnormal apoptosis in lymphocytes; enhancing natural killer cell cytotoxicity; stimulating histamine release from effector cells; enhancing cytotoxic lymphocyte activities and the phagocytic function of macrophages [66,101,126].

In particular, chlorogenic acid, iridoid glycosides, luteolin, lonijaposides A-C, iridoids, 4,5-*O*-dicaffeoylquinic acid, chrysoeriol, japonicaside E, lonijapoglycol A, 1-oxo-(1H)-cyclopenta[b]benzofuran-7-carbaldehyde, 3,5-*O*-dicaffeoylquinic acid, secologanic acid, apigenin, caffeoylquinic acids, loniceralanside, kaempferol, ferulic acid, iridoid glycosides, luteoside, lonicerin, neochlorogenic acid, chrysoeriol, seleno-polysaccharides, uronic acid, secosesquside, japopenoid D, quercetin, kaempferol, isochlorogenic acid-rich fractions, biflavonoid-rich fractions, swertiamarin, isochlorogenic acids A and C, loniceralanside A, ochnaflavone, grandifloroside, and polysaccharides have demonstrated immunomodulatory activities through multiple mechanisms. These include lowering body temperature; decreasing serum inflammatory factors and cytokines, including Bcl-2, p-c-Fos, AP-1, cAMP, prostaglandin E2, inducible nitric oxide synthase (iNOS); enhancing the expression of anti-inflammatory cytokine IL-10; improving the activity of immune enzymes in serum; decreasing goblet cell numbers; maintaining gut microbiota balance; regulating immunity-related gene expression; enhancing total antioxidant capacity and SOD levels in lymphoid organs; increasing apoptosis; lowering serum immunoglobulins levels (IgE, IgM and IgG); decreasing cell migration number and adhesion; upregulating skin barrier-related proteins; promoting the recovery of epithelial barrier function; ameliorating metabolic disturbances (glycerophospholipid metabolism, citrate cycle, arginine biosynthesis, protein digestion and absorption, and nucleotide metabolism); inhibiting hemolysin and protease production and biofilm formation; reducing lymphocyte, eosinophil and neutrophil infiltration; accelerating Ca2+ clearance in lung; restoring the mechanical barrier function of microglial cells and macrophages; inhibiting the release of β-glucuronidase from polymorphonuclear leukocytes; suppressing splenic Th17 cell differentiation; reducing NOD-like receptor family pyrin domain-containing protein 3 (NLRP3) inflammasome activation; inhibiting enzymatic activities of containing cyclooxygenase-2 (COX-2), myeloperoxidase (MPO), 5-lipoxygenase, and matrix metalloproteinase-9 (MMP-9); increasing ubiquitinated proteins; as well as attenuating the interaction between transforming growth factor-β-activated kinase 1 (TAK1) and Kelch-like ECH-associated protein 1 (KEAP1) [83,85,103,160,162,163,164,165,166,167,168,169,170,171]. These immunomodulatory effects have been reported in experimental models involving rats, soft-shelled turtle (Pelodiscus sinensis), zebrafish, dogs, cats, fish, and children [69,71,122,146,147,148,149,150,151,152,153,154,155,156,157].

**Figure 5:**
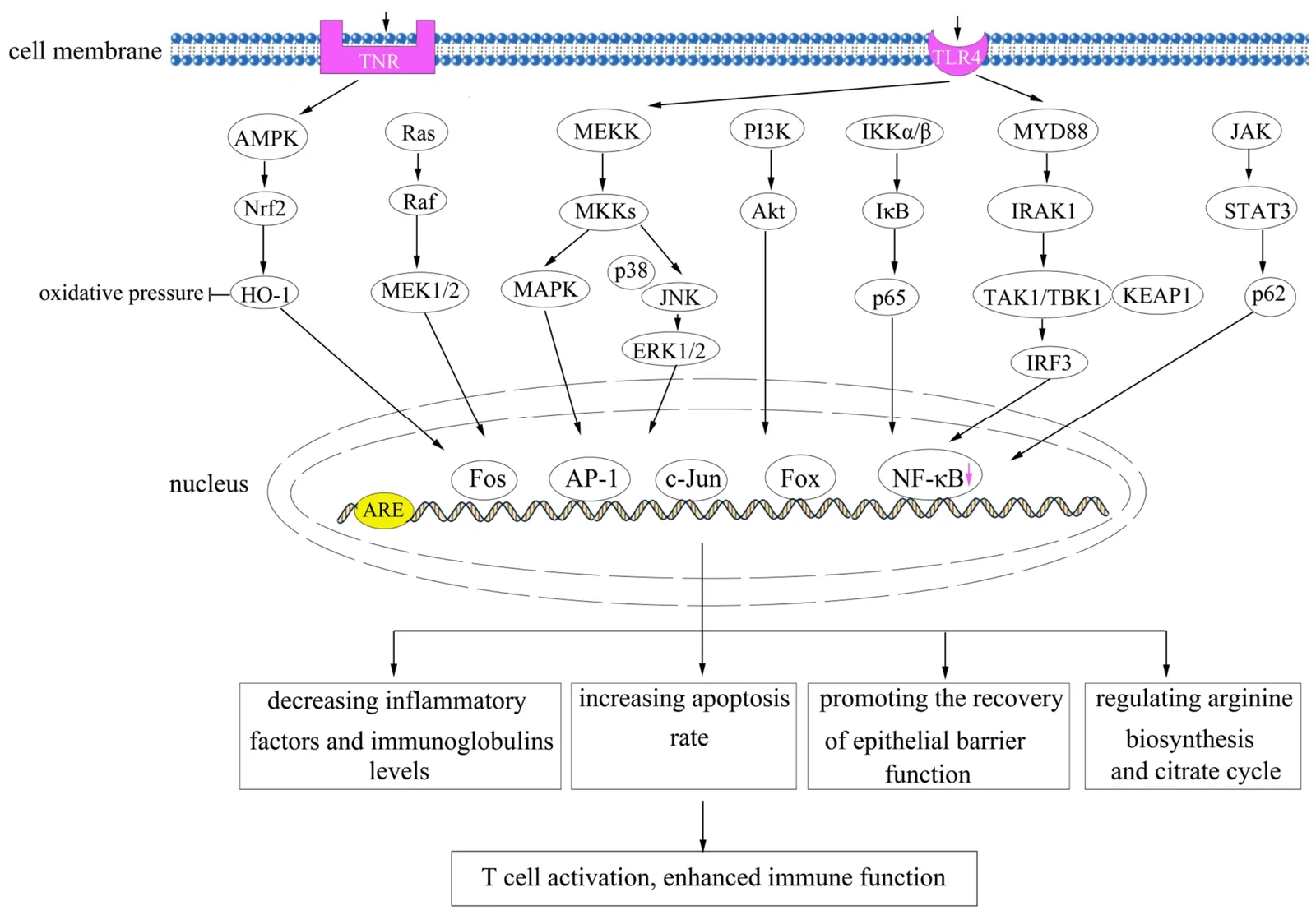
Immunomodulation mechanisms of *L. japonica* active ingredients.

Particularly, active ingredients of *L. japonica* enhanced immune activities via regulating AMPK/Nrf2/HO-1, phosphatidylinositol 3-kinases (PI3K)/Akt/Fox, p62/Janus kinase 1/signal transducer and activator of transcription (STAT)1/3-dependent NF-κB, TLR4/MyD88/IRAK1/TAK1/NF-κB, TBK1/IRF3/NF-κB, IKKα/β-IκB-p65-NF-κB, mitogen-activated protein kinases (MAPKs)/extracellular signal-regulated kinase 1/2/phosphorylation of c-Jun N-terminal kinase (JNK)/p38/c-jun, MAPK/NF-κB/AP-1, and cyclic-GMP-AMP synthase-stimulator of interferon genes pathways (Figure 5) [126,160,172,173,174,175].

### 3.4. Metabolic Regulation

#### 3.4.1. Anti-Obesity

*L. japonica* extracts can effectively improve lipid metabolism by enhancing hepatic pyruvate kinase and hexokinase activities, elevating high-density lipoprotein-cholesterol (HDL-C), alleviating hepatic vacuolar degeneration and nuclear displacement, downregulating the expression of lipogenesis-related genes (*acc1*, *fas*, *srebp1*, and *pparγ*), upregulating genes involved in lipolysis and fatty acid oxidation (*cpt1*, *atgl*, *lpl*, and *PPARα*), lowering lipid accumulation in muscle and liver, reducing plasma triglyceride, as well as reducing retroperitoneal and epididymal adipose tissue in grass carp and mice (Table 4) [132,176]. Polyphenols and polysaccharides were identified as the main active constituents responsible for the prokinetic effects of *L. japonica*, which were manifested by enhanced contractile responses of feline esophageal smooth muscle cells and lower esophageal sphincter muscle strips, as well as accelerated gastric emptying and gastro-intestinal transit [177]. Furthermore, *L. japonica* polysaccharides exerted anti-hyperuricemic activities via decreasing serum uric acid levels and suppressing xanthine oxidase activity [74]. LIPO-700, a standardized herbal formulation combining *L. japonica* and *Taraxacum officinale* F.H.Wigg., exhibited multi-target anti-obesity effects through coordinated regulation of lipid metabolism, suggesting its potential as a safe herbal intervention for obesity management [178].

#### 3.4.2. Anti-Diabetes

*L. japonica* exhibited anti-diabetic effects through multiple mechanisms, including inhibiting and decreasing food and water intake; intestinal α-glucosidase and protein tyrosine phosphatase 1B (PTP1B) activities; improving insulin resistance; restoring body weight; alleviating pancreatic β-islet damage; enhancing the expression of peroxisome proliferator-activated receptor γ (PPARγ) and insulin receptor substrate-1 (IRS-1); increasing SOD activity; decreasing MDA and iNOS levels; enhancing insulin-stimulated glucose uptake; and promoting AKT phosphorylation. *L. japonica* also improved glucose tolerance and dyslipidemia by increasing protective lipid species, including blood polyunsaturated fatty acid-containing diacylglycerol (DAG) species and HDL-C, as well as serum levels of CTRP3/9, while reducing detrimental lipid species, including saturated fatty acid-containing DAG species, LDL-C, and total cholesterol. In addition, it increased the levels of very-long-chain fatty acid-containing ceramide species in blood, upregulated liver PPARa expression and hepatic adiponectin receptors (AdipoR1 and AdipoR2), enhanced downstream AMPK activity, decreased retinal vascular changes, reduced elevated serum vascular endothelial growth factor (VEGF) levels, increased hepatic pyruvate kinase and hexokinase activities, enhanced glycogen accumulation in liver and skeletal muscle, as well as attenuated postprandial hyperglycemia [179,180,181,182,183]. Furthermore, *L. japonica* interacted with α-glucosidase and altered the microenvironment surrounding its amino acid residues, alleviated hepatic oxidative stress, and inhibited *β*-glucuronidase release from polymorphonuclear leukocytes in rats [179,180,181,182,183].

In particular, chlorogenic acid, isochlorogenic acids A, B, and C, neochlorogenic acid, lonjaponspirosides A and B, iridoid glucosides (7-*O*-ethylsweroside, secologanin dimethyl acetyl, adinoside F, *7R*-secologanin *n*-butyl methyl acetal, and adinoside G), coptisine chloride, epiberberine chloride, berberine chloride, jatrorrhizine chloride, and palmatine chloride are major active constituents with significant hypoglycemic and lipid-modulating activities. Their effects were associated with the regulation of the CTRPs-AdipoRs-AMPK/PPARα axis, promotion of ELOVL6 protein expression, and modulation of the phosphorylation of intracellular signaling molecules of NF-*κ*B, p65, I*κ*B *α*, JNK1/2 and ERK1/2 [74,184]. Moreover, *L. japonica*-*Rehmannia glutinosa* mixture (1:1), Korean medicine Yangkyuksanhwa-tang containing *L. japonica* and *Rehmannia glutinosa*, JinQi Jiangtang tablets (Coptidis Rhizoma-Astragali Radix-*L. japonicae*), and refined JinQi-JiangTang tablet (*C. chinensis-Astragalus membranaceus-L. japonica*) all exhibited enhanced antidiabetic and anti-obesity effects [185].

#### 3.4.3. Anti-Cancer

Extracts of *L. japonica* flos, containing various bioactive constituents, including chlorogenic acid, isochlorogenic acids (e.g., isochlorogenic acid C), sweroside, astragaloside, luteolin, rutin, luteoloside, exosomal miR2911, japoflavone D, terpenoids, chrysoeriol, hyperoside, and homogalacturonan-rich pectic polysaccharides, have shown potential anti-cancer activities against pancreatic cancer cells, human papillomavirus (HPV)-associated intraepithelia neoplasia, cervical cancer cells (SiHa, CaSki and HeLa), liver cancer (HepG 2 and SMMC-7721), breast cancer cells (MDA-MB -231), prostate cancer, gastric cancer, myeloid leukemia, and non-small cell lung cancer cells (Table 4 and Table 5). The penicidone E, isolated from the *L. japonica* endophytic fungus *Penicillium* sp. YT2019-3321, showed cytotoxicity against human pancreatic tumor cells PATU8988T with an IC50 value of 11.4 μM [186]. Moreover, *L. japonica* polysaccharides encapsulated in exosomes derived from mouse bone marrow mesenchymal stem cells, *L. japonica* leaves-mediated silver nanoparticles, ethanolic extract of Sophorae flos-*L. japonica*, and the NHE-06 formulation (*Ficus carica*, *Taraxacum mongolicum*, *Angelica sinensis*, *L. japonica*, Pseudo-ginseng and *Folium ginkgo*) demonstrated enhanced anti-cancer activities [69,187].

**Figure 6:**
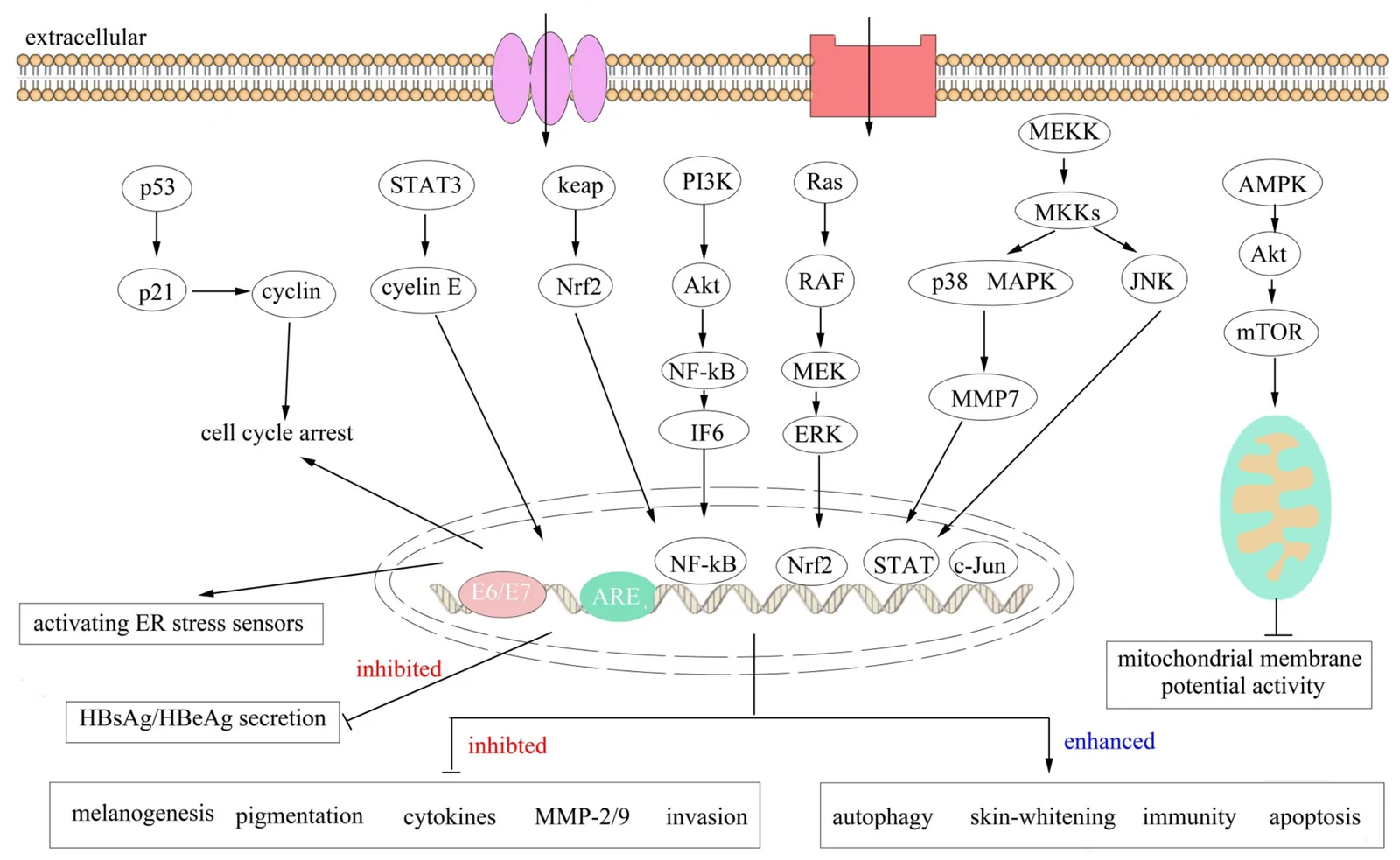
Anti-cancer molecular mechanisms of *L. japonica* active ingredients.

*L. japonica* bioactive ingredients exerted anti-cancer activities through multiple mechanisms, including activation of ER stress sensors; induction of autophagy and cancer cell apoptosis; inhibition of mitochondrial membrane potential activity, reduction in tumor volume and weight; enhancement of Th, Tc and dendritic cell populations in melanomas and spleens; induction of cell-cycle arrest; suppression of cancer cell migration and invasion; inhibition of E6/E7 oncogene expression; reduction in cell viability; modulation of miRNAs including miR-34a, miR-let-7a/f, and miR-301; regulation of poly-(ADP-ribose) polymerase and the B-cell lymphoma-2-associated X-protein/B-cell lymphoma-extra large ratio; enhancement of tumor-reactive CD8+ T cell responses; modulation of receptor tyrosine kinase ligands, inhibition of HBsAg/HBeAg secretion and HBV DNA replication; inhibition of protein kinase B activation by dephosphorylation; upregulation of heme oxygenase-1; reduction in C-C chemokine receptor type 7 (CCR7) expression; and downregulation of cytokines such as Cyclin D1, CDK4, CDC2, CDC25, VEGF, EGF, IL-10, TGF-*β*, and CD34 in splenic lymphocytes. These effects involved multiple signaling pathways, including the E6/E7-p53/Caspase3, KEAP1/NrF2/ARE axis, p38/JNK, PI3K/AKT/mTOR, EGFR/phospholipase Cγ/ERK½/slug, p53/p21, MAPK/ERK, and NF-κB/IL-6/STAT3 pathways (Figure 6) [71,188,189,190,191,192,193]. Moreover, *L. japonicae* active constituents inhibited potent melanogenesis and cellular pigmentation via downregulating tyrosinase and tyrosinase-related proteins in melan-a cells, suggesting skin-whitening effects [150].

**Table 5:** Anti-cancer activity of active ingredients of *L. japonica*.

Components	Types of Cancer	Cell Lines	Activities	Reference
homogalacturonan-rich pectic polysaccharide	hepatoma	hepatocellular carcinoma cell	inhibiting cellular proliferation and migration	[69]
polysaccharide	pancreatic cancer	BxPC-3, PANC-1	inhibiting cells growth	[77]
sweroside	acute myeloid leukemia	human leukemia cell lines	reducing cell viability; inducing S and G2/M cell-cycle arrest; inducing apoptosis	[190]
luteolin	gastric cancer	gastric cancer cells	upregulating miR-34a; downregulating the target HK1protein; inducing G1 phase arrest	[149]
water-decocting extract from *L. japonica*	hepatoma	hepatocellular carcinoma cell	suppressing NF-κB/IL-6/STAT3 signaling	[191]
isochlorogenic acid c	breast cancer	MDA-MB-231	up-regulating epithelial markers; down-regulating mesenchymal markers, decreasing activities of MMP9; inhibiting cell invasion	[135]
japoflavone D	hepatoma	Hepatoma cells	inhibiting cell viability; lessening the damage caused by excess ROS; inducing apoptosis	[153]
terpenoids	liver cancer	HepG 2 and SMMC-7721	inhibiting HBsAg and HBeAg secretion, and HBV DNA replication	[192]
ethanolic extract of Sophorae flos and *L. japonicae* flos	lung cancer	B16F10 and A375	upregulating levels of miR-let-7a/f, lowering protein level of C-C chemokine receptor type 7 (CCR7), inhibiting phosphorylation of CCR7 downstream molecules p38 and JNK, lowering levels of MMP-2 and MMP-9	[134]
extract from *L. japonicae*	breast cancer	4T1, EMT6, BT-549, and MDA MB-231	inhibiting cell viability; reducing tumor volume and weight; inducing apoptosis	[193]
leaves-mediated silver nanoparticles	breast cancer, cervical cancer, liver cancer	HepG2, MDA-MB -231, and Hela cells	inhibiting potent melanogenesis; inhibiting body pigmentation; enhancing skin-whitening; decreasing tyrosinase, tyrosinase-related protein-1 (TRP-1) and TRP-2 protein production	[116]
penicidone E isolated from *Penicillium* sp. (*L. japonica* endophytic fungus)	pancreatic tumor	PATU8988T	improving morphologies abnormity; upregulating proportion of apoptotic cells; elevating the ratio of Bcl-2 to Bax	[194]
exosomal miR2911	HPV16/18-positive cervical cancer	SiHa, CaSki and HeLa	suppressing RNA and protein expression of E6/E7 oncogenes, inducing cell apoptosis	[195]
polysaccharides encapsulated into exosomes	tumor-reactive T cell	CD8+ T cell	enhancing tumor-reactive cell responses; building patients’ immunity; causing prophylactic tumor inhibition	[196]
flower bud extracts	lung cancer	NSCLC	upregulating PECAM1 and SPP1 expression	[197]

### 3.5. Benefits for Cognitive Function

*L. japonica* extracts and their bioactive constituents, including galuteolin, chlorogenic acid, and water-soluble polysaccharides, have demonstrated neuroprotective effects in experiment model of Alzheimer’s disease, sleep disorders, depression, cerebral ischemia/reperfusion injury, neuroinflammation, and cerebral infarction (Table 4). These effects were associated with reduced neuron damage in the hippocampal carotid artery 1 pyramidal layer, increased neuron-specific enolase levels, decreased expression levels of autophagy-related proteins, reduced brain water content, enhanced antioxidant capacity during cerebral ischemia/reperfusion injury, alleviation of pathological changes in the cerebral cortex, and decreased levels of pro-inflammatory cytokines (IL-6, TNF-α, and IL-1β) in serum and the hippocampus. *L. japonica* extracts also inhibited microglial and NLRP3 inflammasome activation, attenuated hippocampal neuron damage, inhibited Aβ42 aggregation and oligomerization, prevented fibril formation, promoted neuritogenesis, and modulated hypoxia-related factors [72,76,194,195,196]. Moreover, *L. japonicae* extracts alleviated sleep deprivation by reducing sleep latency and increasing non-rapid eye movement sleep during daytime and rapid eye movement sleep during recovery from acute sleep deprivation [139]. Particularly, chlorogenic acid and polysaccharides existing in *L. japonica* flower extracts exhibited significant antidepressant effects.

Moreover, *L. japonica* flower extracts could prevent hippocampal amyloid-β accumulation, enhance hippocampal insulin signaling, decrease TNF-α and iNOS levels in the hippocampus, alleviate cognitive dysfunction and neuroinflammation and prevent impairment of energy and glucose homeostasis in rats [197]. In addition, *L. japonica* flower extracts exhibited potential against Alzheimer’s disease through multi-component and multi-target interactions, with lignan interacting with epidermal growth factor receptor (EGFR); β-carotene with catenin beta-1 and apoptosis regulator Bcl-2; β-sitosterol with apoptosis regulator Bcl-2; hederagenin with nuclear factor NF-kappa-B p105 subunit; berberine with EGFR and Bcl-2; and baicalein with NF-kB p105, EGFR, and Bcl-2 [195].

### 3.6. Benefits for Bone and Skin Health

Fermented and unfermented *L. japonica* extracts have been shown to improve bone mineral density, restore bone microarchitectural damage, increase serum calcium and osteocalcin levels, inhibit osteoclast differentiation, promote osteoblast differentiation, reduce bone loss, alleviate joint bone erosion, decrease actin-ring formation and bone-resorbing activity, and restore gut microbial composition and serum metabolites in arthritis rats [198,199]. Moreover, the ethanolic extract of *L*. *japonicae* combined with *Rosae Multiflorae Fructus* showed enhanced bone protective activity [198]. Active ingredients of *L. japonica* extracts, including ChondroT and (*R*)-dehydroxyabscisic alcohol β-D-apiofuranosyl-(1→6)-β-D-glucopyranoside, may promote bone health via regulating STAT/JNK, NF-κB/IκBα/p38/MAPKs, Wnt1/Wnt3/runt-related transcription factor 2, and Smad1/Smad5/Smad8/β-catenin/runt-related transcription factor 2 signaling pathways. These effects were associated with decreased tartrate-resistant acid phosphatase-5b levels, enhanced alkaline phosphatase activity, promotion of human mesenchymal stem cell mineralization, reduction in multinucleated bone marrow macrophages, increased expression of bone differentiation markers and bone morphogenetic proteins, downregulation of osteoclast-specific proteins, and suppression of pro-inflammatory cytokines, including TNF-α, IL-6, IL-17, c-Fos, and IL-1β [100,102,198].

*L. japonicae* extracts exhibited skin protective effects by alleviating collagen disruption and reducing levels of pro-inflammatory cytokines, including TNF-α, IFN-γ, IL-6, IL-17, IL-23, NO, sPLA2, and IF-1β, in dorsal skin against UVB radiation. They also inhibited cell proliferation, induced apoptosis, downregulated VEGF, and inhibited topoisomerase-II activity through modulation of inflammatory, cellular stress and EGFR-related signaling pathways [199]. *L. japonica* bioactive substances exhibited potential anti-psoriatic effects at the cellular level via multiple mechanisms, including antiproliferative, pro-apoptotic, anti-inflammatory, and anti-angiogenic activities [200].

### 3.7. Benefits for Internal Organs

*L*. *japonicae* flos is traditionally associated with the lung, heart, kidney, and stomach meridians and is used for its anti-inflammatory, antimicrobial, antiviral, and antipyretic activities (Table 4).

#### 3.7.1. Hepatoprotective Effect

Ethanol extracts of *L. japonica* have demonstrated hepatoprotective effects in various experimental models of nonalcoholic steatohepatitis, sepsis-induced liver injury, liver fibrogenesis, alcoholic liver injury, alcohol-induced addiction, hepatic steatosis, and other forms of hepatic inflammation. These effects are associated with improvement in body weight and daily food intake; reduction in hepatic lipid accumulation and serum lipopolysaccharide levels, promotion of anti-inflammatory macrophage activity; attenuation of hepatic inflammation via regulating expressions of genes involving in immune response; enhancement of antioxidant ability; restoration of gut microbiota homeostasis; reduction in lipid droplet vacuolization and inflammatory cell infiltration, increased cell viability and inhibition of hepatic stellate cells activation; attenuation of apoptosis; increased metformin concentration in liver; improved glucose tolerance activity; reductions in serum markers of liver damage indices, including alanine aminotransferase, aspartate aminotransferase, and alkaline phosphatase; downregulation of hepatic peroxisomal acyl-CoA:diacylglycerol acyltransferase 2 expression; amelioration of hepatocyte balloon degeneration; attenuation of alcohol-induced abnormal enhancement of addiction memory; decreased LDH release, reduced deposition of collagen and alpha-smooth muscle actin; enhanced intestinal permeability, and reversal of the epithelial–mesenchymal transition process in rodents [13,38,129,201,202]. Particularly, hyperoside, isochlorogenic acid B, hederagenol, 3-caffeoylquinic acid, 4-caffeoylquinic acid, 5-caffeoylquinic acid, 3,5-dicaffeoylquinic acid, 4,5-dicaffeoylquinic acid, luteolin-7-*O*-glucoside, protocatechuic acid, sweroside, quercetin, kaempferol, luteolin, and secoxyloganin have been reported as the main active ingredients associated with hepatoprotective effects. Their activities involve regulation of the PPAR/NF-κB, autophagy, Nrf2/MAPK/JNK, and FXR-miR-29a signaling pathways [203,204]. BaWeiBaiDuSan, containing *L. japonica* extract and a fermentation broth of a mixture (*Pueraria lobata*, *L. japonica*, and *Crataegus pinnatifida*) by *Lactobacillus rhamnosus* 217-1, has also demonstrated enhanced hepatoprotective effects [205].

#### 3.7.2. Benefits for Lung Health

*L. japonica* extracts can mitigate particulate-matter 2.5 (PM2,5)-induced pulmonary inflammation, fibrosis, lung injury, and ferroptosis through regulating the TGF-β/NF-κB, IL-17 signaling pathway, and Nrf2/GPX4 signaling pathways [130,142,206,207,208]. Its main active ingredient, including luteolin, luteolin-7-*O*-rutinoside, apigenin-7-*O*-rutinoside, amentoflavonem, shanzhiside, secologanoside, loganic acid, chlorogenic acid, secologanic acid, secoxyloganin, quercetin pentoside, and dicaffeoyl quinic acids, exhibited pulmonary protective effects by reducing accumulation of pro-inflammatory cytokines (TNF, IL-1, and IL-6), ROS, MDA, and Fe^2+^; modulating T cell populations (CD4^+^ T, CD8^+^ T, and Th2 cells); increasing levels of anti-inflammatory cytokines; restoring the balance of Th1/Th2/Th17/Treg cells in the lungs and colons; alleviating pulmonary pathological injury; restraining apoptosis via protecting matrix Metalloproteinases; decreasing the levels of oxidative stress factors and protecting pulmonary antioxidant system; and ameliorating mitochondrial function by regulating mitochondrial membrane potential and ATP contents. These effects collectively contributed to the alleviation of acute lung injury in mice [130,206,207,208].

#### 3.7.3. Benefits for the Cardiovascular System

*L. japonica* extracts exerted profound cardioprotective effects and prevented abdominal radiotherapy-induced vascular endothelial dysfunction by disrupting capillary-like tube formation of human microvascular endothelia cells, inhibiting cell migration through decreasing expression of Notch1 and Delta-like ligand 4, reducing pro-inflammatory factor levels, oxidative stress, and superoxide anion production; resisting leukocyte infiltration, reducing platelet-monocyte aggregation, and platelet–leukocyte interactions; suppressing platelet-granulocyte aggregation; improving vascular remodeling, increasing mitochondrial membrane potential; inhibiting cardiomyocytes apoptosis; regulating vasodilatory action; increasing NO levels; restoring mesenteric arterial blood perfusion; enhancing cardiac contractile function; ameliorating cardiac pathological remodeling; regulating glucose and lipid metabolism; and attenuating inflammation in mice with heart failure [64,209]. These active ingredients, particularly polysaccharide and chlorogenic acid, exerted profound cardioprotective effects and circulatory guarding properties via regulating GTPCH1/BH4/eNOS, calmodulin-dependent protein kinase II delta/NF-κB, Raf/MAPK/ERK, and NF-κB/p65/JNK/ERK1/2 pathway [210,211]. In addition, the combination of *L*. *japonicae* with *Angelicae sinensis* Radix showed enhanced cardioprotective activities [209]. Moreover, caffeoylquinic acids also possessed potent myocardial protective activity in a dose-dependent manner against ischemic heart diseases, primarily through relieving oxidative stress [212].

#### 3.7.4. Gastroprotective Effect

Aqueous extracts of *L. japonica* exerted gastroprotective effects by reducing gastric lesions, enhancing gastric mucus secretion and antioxidant activities, suppressing increases in pro-inflammatory mediators (TNF-α, NF-κB, IL-6, and IL-1β), and increasing the levels of hexosamine, sialic acid, and prostaglandin E2 in gastric mucus [143].

## 4. Applications in the Food Industry

*L. japonica* has been widely used as a food ingredient and in tea and other beverages, including *L. japonica* tea (prepared by combining *L. japonica* with black, yellow, or green tea), *L. japonica*-based nutritional beverages, *L. japonica* acidophilus milk, *L. japonica* soup, *L. japonica* wine, and *L. japonica* oral liquids [141]. *L. japonica* flos has also been developed into dried tea using four main fixation methods, including sun drying, hot-air oven drying, stir-frying, and steaming [44]. In addition, *L. japonica* extracts can decrease lightness, increase redness, reduce hardness, increase crispiness, and enhance the antioxidant activity of cookies [146]. Moreover, *L. japonica* extracts, especially essential oil extracted from its flower buds, serve as preservatives for typical long-term storage and can even withstand the traditional deep-frying processes [156]. In particular, the addition of *L. japonica* essential oil significantly improved the oxidative stability of sunflower oil by hindering the reduction in acidity value, peroxide value, p-anisidine value, total oxidation value, and the levels of thiobarbituric acid reactive substances and total polar compounds [156]. *L. japonica* extract can also serve as a ruminal fermentation modifier to suppress methane production with minimal effects on nutrient digestion in the rumen.

*L. japonica* extracts can increase dry matter intake and milk yield, improve milk composition, reduce rectal temperature and serum creatinine levels, improve rumen fermentation, increase serum triiodothyronine and thyroxine concentrations, and reduce milk somatic cell counts and circulating biomarkers of pro-inflammatory responses in high-yielding dairy cows [136,137,138]. In particular, organic acids such as chlorogenic acid, polysaccharides, flavonoids and terpenoids were identified as the main contributors to the anti-inflammatory effects of *L. japonica* extract. In addition, a herbal extract mixture (0.05–0.10%) comprising *Scutellaria baicalensis* and *L. japonica* can support overall health and enhance growth performance in growing-finishing pigs under hot and dry climatic conditions [213].

## 5. Prospects and Challenges

*L. japonica* has been widely appreciated as a food and beverage owing to its biological activities, characteristic aroma and flavor. *L. japonica*–based products have attracted increasing interest and popularity in recent years. They include tea infusions, nutritive beverages, acidophilous ‘milk’ (fermented emulsion), soups, wine, and oral liquid among others. In particular, organic acids, mainly chlorogenic acid, as well as polysaccharides, flavonoids and terpenoids, are considered main contributors serving as effective ingredients responsible for the anti-heat stress properties of *L. japonica* extract. Processing can alter the properties and functions of *L. japonica*, potentially increasing efficacy while reducing toxicity and adverse effects. Moreover, *L. japonica* exhibits multi-component, multitarget, and multi-pathway actions in the management of metabolic diseases.

Significant differences in chemical composition have been observed among flowers, flower buds, stems, and leaves of *L. japonica*, and these differences may contribute to their distinct pharmacological activities. Therefore, different medicinal parts of *L. japonica* should be appropriately selected according to the intended functional application. Cultivation practices may also affect its phytochemical composition and health-promoting properties. In addition, adulteration and substitution of *L. japonica* with low-quality materials are relatively common. Existing primers and TaqMan probes developed based on the 5.8S-ITS2 rDNA sequence have shown limitations in distinguishing *L. japonica* from closely related species. Research on tissue culture systems for *L. japonica* remains limited, which may constrain its potential for enhancing secondary-metabolite production and alleviating resource shortages. Moreover, pesticide residues, including carbendazim, chlorbenzuron, imidacloprid, chlorpyrifos, and acetamiprid, in *L. japonica* have attracted considerable attention because of their potential cumulative health risks, particularly for children and other susceptible populations. Processing may alter the properties and functions of remedies, enhancing medicinal effects while decreasing toxicity and adverse effects. Furthermore, the oral bioavailability of phenolic acids in beverages prepared from *L. japonicae* is also relatively low.

As demand for *L. japonica* continues to increase, the development of superior cultivars with improved quality is imperative. This will require the establishment of germplasm resource banks and mutant libraries, development of efficient genetic transformation systems, identification and characterization of key functional genes, as well as development of molecular markers closely linked to these genes. The adulteration and substitution of *L. japonica* with lower-quality materials highlights the need for reliable and sensitive methods for authenticating *L. japonica* flos to ensure consumer safety. Further research is needed to elucidate the regulation of secondary metabolite biosynthetic pathways, optimize the scientific use of pesticides in large-scale cultivation, improve processing technologies, clarify the underlying mechanisms and in vivo effects in animal models, and evaluate food supplements or edible excipients as potential enhancers of intestinal tight junction integrity. Collectively, these efforts will provide a scientific foundation for the sustainable utilization and clinical and non-clinical applications of *L. japonica*.

## Figures and Tables

**Table 1:** Polysaccharides isolated from *L.japonica*.

Section	Molecular Weight (Da)	Components	Activities	References
CLJP	1450	galacturonic acid (23.57%), galactose (23.45%) and arabinose (23.45%)	anti-aging	[63]
LJP-2-1	1280	galacturonic acid (51.25%) and arabinose (22.89%)	anti-aging	[63]
LJW2F2	7.2 k	α-1,4-D-galacturonic acid	disrupting angiogenesis	[64]
PHL	137.97 ± 4.31 k	glucose (47.40 mol%), galactose (19.21 mol%) and arabinose (20.21 mol%)	enahncing protective effect on β-carotene against UV irradiation	[65]
LJP-N	5.4 k	starch-like glucan as well as some arabinogalactan and/or arabinan domains	immunomodulatory effect	[66]
LJP-A	400 kDa	galacturonan with some galactan and/or arabinan domains	immunomodulatory effect	[66]
WLJP-A0.2b	40.6 k	rhamnogalacturonan I (branched α-L-1,5-arabinan, β-D-1,4-galactan and type II arabinogalactan side chains): rhamnogalacturonan II: homogalacturonan (un-esterified and partly methyl-esterified and/or acetyl-esterified oligogalacturonides with degree of polymerization 1-8) domains = 0.4:1.0:2.1	anti-oxidation activity	[67]
LJCP-2b	7.0 k	glucose, galacturonic acid, galactose, arabinose, rhamnose, xylose, mannose and glucuronic acid	inhibiting hyperproliferation and inflammatory response of rheumatoid arthritis fibroblast-like synoviocytes	[68]
WLJP-03A	28.1 kDa	Rha (5%), GalA (60%), Gal (5%), and Ara (30%)	modulating galectin-4-mediated bioactivity and anti-hepatocellular carcinoma activity	[69]
HEP-4	1.98 × 10^5^ Da	mannose, rhamnose, galacturonic acid, glucose, galactose, and arabinose with a mole ratio of 6.74:1.56:1.04:14.21:4.31:5.4	antioxidant activities	[70]
LJP	1–1000 kDa	8.7% GalA, 8.2% Rha, 16.2% Gal, 19.5% Ara, 26.9% Glc and 20.5% Man	strong protective effect on mice with depression	[71]
LFA03-a	-	rhamnose, arabinose, galactose and galacturonic acid in the molar ratio of 18.1:25.3:36.8:19.5	antioxidant activities	[72]
LJP-N	19.0–383.8 kDa	starch-like glucan with some arabinogalactan	antioxidant activities	[73]
LJP-A	19.0–383.8 kDa	GalA (>50%) with some Gal and Ara residues	antioxidant activities	[73]
LJP-1	17.5 kDa	glucuronic acid, glucose, galactose, arabinose, and xylose at the ratio of 2.43:1:2.09:1.95:1.96	anti-hyperuricemic and anti-gouty arthritis	[74]
LJLP	-	galactose (32.3%), glucose (20.9%), and ribose (15.2%)	antioxidant activity	[75]
LJPB2	8.9 × 10^3^ Da	arabinose, mannose, glucose, and galactose, in a molar ratio of 1.8:1.0:3.6:3.7	neuroprotective effect on cerebral ischemia–reperfusion injury	[76]

**Table 2:** Antimicrobial properties of *L. japonica* active ingredients.

Activities	Active Component	Microorganism	Minimum Inhibitory Concentration (MIC)	Reference
Antiviral activities	chlorogenic acid	respiratory syncytia virus	0.05 mg/mL	[2]
	chlorogenic acid	coxsackie B3 virus	0.1 mg/mL	[2]
	chlorogenic acid	adeno-associated 7 viruses	0.4 mg/mL	[2]
	chlorogenic acid	adeno-associated 3 viruses	0.8 mg/mL	[2]
	chlorogenic acid	Coxsackie B5 virus	0.8 mg/mL	[2]
	chlorogenic acid	human cytomegalo virus	1 μg/mL	[2]
	chlorogenic acid	*Mycoplasma gallisepticum*	-	[87]
	chlorogenic acid	H1N1, H3N2, and oseltamivir-resistant strains	-	[88]
	methyl caffeate	Coxsackie virus B3	3.70 μmol/L	[89]
	2′-*O*-methyladenosine	Coxsackie virus B3	6.41 μmol/L	[89]
	*Lonicerae japonicae* flos-*Fructus forsythiae* herb chito-oligosaccharide	influenza H1N1 virus	-	[90]
	Honeysuckle-Huangqi	SARS-CoV-2	-	[91,92]
	Shuang-Huang-Lian injectable powder (*Scutellaria baicalensis*, *L. japonica*, and *Forsythia suspense*)	human Adenovirus 3		[93]
Antibacterial activities	7-*O*-ethyl sweroside, secologanin dimethylacetal, adinoside F, (7R)-secologain n-butyl methyl acetal and adinoside G	*Staphylococcus aureus* ATCC 25923	from 13.7 to 26.0 µg/mL	[74]
	chlorogenic acid	*Aeromonas hydrophila*	6.4 mg/mL	[94]
	chlorogenic acid	*Staphylococcus aureus*	0.8 g/mL	[2]
	chlorogenic acid	*Escherichia coli*	0.025 g/mL	[2]
	chlorogenic acid	shigella	0.125 mg/mL	[2]
	chlorogenic acid	salmonella	0.125 mg/mL	[2]
	chlorogenic acid	*Sarcina luteus*	0.025 g/mL	[2]
	50% ethanol extract; ethyl acetate fraction	*Staphylococcus aureus*	-	[95]
	chlorogenic acid; ethyl acetate fraction	*Bacillus subtillis*	0.1 g/mL	[2,95]
	50% ethanol extract; ethyl acetate fraction	*Pseudomonas aeruginosa*	-	[95]
	water extracts	*Bacillus cereus*	-	[96]
	ethanolic extract	*Bacillus cereus*		[96]
	luteolin	*Bacillus cereus*	15.625 μg/mL	[96]
	quercetin	*Bacillus cereus*	31.25 μg/mL	[96]
	kaempferol	*Bacillus cereus*	15.625 μg/mL	[96]
	AgNPs-HWE	*Escherichia coli* CMCC44113		[97]
	*L. japonica* extract	*Streptococcus mutans*	40 mg/mL	[98]
	7-acetyl-8,9-dihydroxy thymol	*Staphylococcus aureus*	78.52 ± 1.37 µg/mL	[99]
	7-acetyl-8,9-dihydroxy thymol	*Escherichia coli*	27.64 ± 2.26 µg/mL	[99]
	7-acetyl-8,9-dihydroxy thymol	*Micrococcus luteus*	63.70 ± 4.41 µg/mL	[99]
	7-acetyl-8,9-dihydroxy thymol	*Bacillus cereus*	52.45 ± 5.53 µg/mL	[99]
	7,8-dihydroxy-9-buyryl thymol	*Staphylococcus aureus*	108.14 ± 9.50 µg/mL	[99]
	7,8-dihydroxy-9-buyryl thymol	*Escherichia coli*	77.63 ± 6.24 µg/mL	[99]
	7,8-dihydroxy-9-buyryl thymol	*Micrococcus luteus*	128.58 ± 13.26 µg/mL	[99]
	7,8-dihydroxy-9-buyryl thymol	*Bacillus cereus*	50.31 ± 8.43 µg/mL	[99]
Antifungal activities	ethyl acetate fraction	*Candida albicans*	-	[95]

**Table 3:** Antioxidant activity of *L. japonica* active components.

Components	Antioxidant Property	Test Model	Reference
methanol/acetone extracts	tyrosinase inhibition, xanthine oxidase inhibition, and nitrite scavenging activities	hepatocyte L-02	[2]
flavone hyperoside	decreasing ALT, AST and MDA, and increasing GSH and cell survival rate	hepatocyte L-02	[2]
de-esterified oligogalacturonides	reducing reactive oxygen species production	HEK-293T cells	[67]
water-soluble polysaccharide (HEP-4)	inhibiting oxidative damage, reducing MDA levels, inhibiting ROS production, enhancing activities of GSH-Px and CAT, and increasing the activity of cells	HepG2 cells	[70]
caffeoylquinic acids	protecting cardiomyocytes from necrosis and apoptosis via attenuating ROS production, reducing the ratio of GSSG/GStotal, activating the caspase-3 apoptotic pathway (Bcl-2, Bax, and caspase-3)	cardiomyocytes	[123]
CLJPs	prolonging mean lifespan by 13.97%, promoting fitness with increased motility by 40.92% and pharyngeal pumping by 25.72%, activating stress-related genes and the antioxidant system, and decreasing lipofuscin accumulation by 38.9%	*Caenorhabditis elegans*	[63]
polysaccharides	improving the activity of SOD, CAT, GSH-Px; decreasing MDA level in serum, liver and brain; restoring the content of AChE in brain; reversing liver tissue damage; enhancing hazardous substances degradation and flavone and flavonol biosynthesis	Aging ICR mic	[114]
LJ-E (500 μg/mL)	enhancing the mean lifespan and significantly improved age-associated physiological functions	*Caenorhabditis elegans*	[99]
chlorogenic acid (54 μg/mL), 1,5-dicaffeoylquinic acid (15 μg/mL), and 1,3-dicaffeoylquinic acid (7.5 μg/mL)	delaying paralysis caused by Aβ toxicity	*Caenorhabditis elegans*	[99]
LJPB2	scavenging free radicals, improving neurological deficit scores and infarct volume, reducing MDA level and NO production, and elevating SOD and GSH-Px enzyme activities	Brain tissues	[76]
4,5-di-*O*-caffeoylquinic acid methyl ester	resisting oxidative stress, attenuating oxidative damage	HepG2 cells	[124]

**Table 4:** Protective effects of *L. japonica* active components in animal models.

Components	Model	Dose	Activity	Reference
polysaccharides extraction from *L. japonica* leaves	male nude Kunming mice aged 30 days	100, 200, 400, and 800 mg/kg, BW per day for 6 weeks	antioxidant activity	[75]
Acidic component of *L. japonica* polysaccharides	sixty female BALB/c mice (20–22 g, 6–8 weeks)	200 mg/kg/d	immunomodulatory effect	[66]
purified water-soluble polysaccharide	male (SD rats weighing (6–8 weeks, 200 ± 20 g)	100, 200, 300 mg/kg	anti-hyperuricemic and anti-gouty arthritis activities	[74]
ethyl acetate extracts of *L. japonica* (ECLJ), n-BuOH extracts of *L. japonica*	male ICR mice (18–22 g), adult male SD rats (200–230 g)	0.42 g/kg for ECLJ; 0.42 g/kg for BCLJ	anti-inflammatory efficacy	[83]
polysaccharide	mice (20.4 ± 1.5 g, 8–12 weeks)	50–150 mg/kg, for 15 days	improving ulcerative colitis	[113]
polysaccharides	ICR mice	50–100 mg/kg/day for 8 weeks	alleviating oxidative stress and anti-aging	[114]
powder of FLJ extract	30 six-week-old female BALB/c mice	0.4–400 mg/kg, once	immunomodulatory effect	[125]
polysaccharide	mice	30, 60 mg/kg body weight	alleviating atopic dermatitis	[126]
LJP	24 mice	800 mg/kg polysaccharide for three days	immunomodulatory	[127]
ethyl acetate fraction of dried flower buds	BV-2 Microglial Cells		inhibiting inflammatory responses	[128]
ethanol extract	C57BL/6 J mice	100–300 mg/kg/day for four weeks	regressing nonalcoholic steatohepatitis	[129]
ethanol extract	13 male BALB/c mice (8 weeks old)	20–100 mg/kg, once a day for 12 weeks	improving pulmonary inflammation, apoptosis, and fibrosis	[130]
*L. japonica* and Radix Puerariae crude extracts	72 healthy Duroc × Landrace × Yorkshire crossbred pigs (93 ± 2 kg)	0.5–1 kg/ton crude extract	regulating metabolism	[115]
*L. japonica* water extracts	three hundred fish (72.50 ± 8.45 g)	10, 50, or 100 g/kg diet for 10 weeks	enhancing growth and non-specific immune responses	[131]
substitutes of *L. japonica*	diabetic rats	800 mg/kg for 42 days	hypoglycemic effects	[132]
crude polysaccharides	diabetic rats	800 mg/kg body weight LJP for 42 days	hypoglycemic and hypolipidemic effects	[133]
formula comprising Sophorae Flos and *L. japonica* Flos	18 eight-week-old male C57/BL6 mice	0.6, 1.2, 2.4 g/kg	anti-metastatic effects	[134]
ethanolic extract of Sophorae flos and *L. japonica* flos	male nude Kunming mice aged 30 days	1.2 g/kg	inhibiting tumor growth	[134]
sweroside	zebrafish	300 μM	inhibiting melanogenesis; inhibiting body pigmentation; enhancing skin-whitening	[135]
*L. japonica* extract	24 healthy Chinese Holstein mid-lactation dairy cows	28 g/d for 10 weeks over a hot summer	enhancing lactation performance, antioxidant status, endocrine, and immune function	[136]
LJE-10% CGA	45 healthy Chinese Holstein high-yielding dairy cows	35 g/(d·head)	improving lactation performance, serum parameters, and rumen fermentation	[137]
LJE-20% CGA	45 healthy Chinese Holstein high-yielding dairy cows	17.5 g/(d·head)	improving lactation performance, serum parameters, and rumen fermentation	[137]
extract	eighteen Holstein dairy cows	1 and 2 g LJE for 60 days	improving lactation performance, enhancing anti-inflammatory and antioxidant capacities	[138]
polysaccharide	male KM mice (6–8 weeks old, 20 ± 2 g)	30, 100 mg/kg for 21 days	anti-depression	[71]
water-soluble polysaccharide (LJPB2)	rat with focal ischemia/reperfusion injury in brain	-	neuroprotective effect	[76]
*L. japonica* flos extract	male C57BL/6 J mice (8–12 weeks old; weight: 20–25 g)	4 g/L for 7 days	promoting sleep	[139]
polysaccharide	male Kunming mice (6–8 weeks old) 20 ± 2 g	1 mg/mL and 2 μL	improving learning and memory impairments via regulation of autophagy	[140]
Fermented *L. japonica* solution	30 female SD rats aged 7–8 weeks	1 mL/100 g daily for nine weeks	improving osteoporosis	[141]
*L. japonica* flos-Forsythiae Fructus	36 female C57BL/6 J mice (6–8 weeks old)	LJF (1.6 g/kg) + FF (1.6 g/kg)	protection against acute lung injury	[86]
*L. japonica* extract	6–8-week-old male C57BL/6 mice (20–30 g)	LTE (1.75 g/kg) for 7 days	ameliorating acute lung injury	[142]
luteolin	6–8-week-old male C57BL/6 mice (20–30 g)	18, 35, 70 µmol/kg for 7 days	ameliorating acute lung injury	[142]
water extract	rat with gastritis and gastric ulcer	50, 100, or 200 mg/kg	gastroprotective effects	[143]

## Data Availability

No new data were created in this article.

## Abbreviation

ACE2	angiotensin-converting enzyme 2
CPLJ	crude polysaccharides from *L. japonica*
DAG	diacylglycerol
ER	endoplasmic reticulum
HO-1	heme oxygenase-1
IFN	interferon
iNOS	inducible nitric oxide synthase
JNK	c-Jun N-terminal kinase
LJ-02-1	a homogeneous polysaccharide extracted and purified from flowers of *L. japonica* by DEAE-cellulose and Sephacryl S-200HR column
LJP	*L. japonica* polysaccharides
MAPKs	mitogen-activated protein kinases
MDA	malondialdehyde
MMP	matrix metalloproteinase
NO_3_^−^	nitrate
NH_4_^+^	ammonium
NLRP3	NOD-like receptor family protein 3
PI3K	phosphatidylinositol 3-kinases
ROS	reactive oxygen species
SARS-CoV-2	severe acute respiratory syndrome coronavirus-2
SOD	superoxide dismutase
STAT	signal transducer and activator of transcription
TAK1	transforming growth factor-β-activated kinase 1
TRP	tyrosinase-related protein
